# Editorial: Self-harm behaviors from young adults to the elderly: prevalence, prevention and treatment following the COVID-19 pandemic

**DOI:** 10.3389/fpsyt.2023.1331376

**Published:** 2023-11-30

**Authors:** Aida Jaffar, Sherina Mohd Sidik, M. Radzniwan A. Rashid, Halyna Lugova

**Affiliations:** ^1^Faculty of Medicine and Defence Health, Universiti Pertahanan Nasional Malaysia, Sg Besi, Wilayah Persekutuan, Malaysia; ^2^Faculty of Medicine and Health Sciences, Universiti Putra Malaysia, Serdang, Selangor, Malaysia; ^3^Faculty of Medicine and Health Science, Universiti Sains Islam Malaysia, Nilai, Malaysia; ^4^Faculty of Medicine and Health Sciences, UCSI University (Springhill Campus), Port Dickson, Negeri Sembilan, Malaysia

**Keywords:** self-harm behavior, young adult, elderly, COVID, pandemic

## Introduction

The COVID-19 pandemic, which swept across the globe in 2020, not only brought on a public health crisis but also had far-reaching consequences for mental health. As individuals and communities grappled with uncertainties, lockdowns, social distancing measures, and unprecedented loss of lives, the toll on mental wellbeing became increasingly evident. Among the myriad mental health challenges, one concerning trend that emerged was the significant rise in self-harm behaviors.

## Impact of pandemics

The pandemic introduced a range of stressors that affected people of all age groups, socio-economic backgrounds, and walks of life. The fear of infection, loss of loved ones, economic instability, pandemic fatigue, and disruption of daily routines contributed to heightened psychological distress (1–4). Lockdowns and social distancing measures enforced isolation, depriving individuals of their usual social interactions and support systems. When mental health problems were considered in the context of previous pandemic outbreaks, the absence of face-to-face contact with friends and family led to loneliness, a significant factor contributing to self-harm (5). Feelings of isolation can intensify pre-existing mental health conditions and trigger self-harming behaviors as a coping mechanism.

The pandemic increased mental health concerns, straining services (6). Access challenges led to delayed or inadequate treatment, contributing to potential gaps and self-harm behaviors. The elderly, who were at higher risk of severe illness, often experienced heightened feelings of isolation (7). Young adults, particularly college students, grappled with the stress of disrupted education and uncertainty about the future (8). These groups faced unique challenges in maintaining their mental wellbeing. A foundation for understanding the intricate interplay of psychological factors leading to self-harm and suicidal thoughts is essential in addressing the complex realm of cognitive dysfunction associated with suicidal behavior (Seng Choi).

The constant exposure to information and news about the pandemic through digital media was overwhelming. Misinformation, panic-inducing headlines, and the continuous news cycle increased anxiety and made it challenging for individuals to disengage from distressing content. In the face of these stressors, self-harm emerged as a coping mechanism for some individuals. According to recent theorizations, self-harm, whether in the form of cutting, burning, or other behaviors, offers temporary relief from emotional pain, pleasurable pain and a sense of control over overwhelming emotions (9). Self-harm can function as a means to convey emotional distress or inner turmoil that may be challenging to verbalize.

## Assessment tool of self-harm behavior

In the field of mental health, the utilization of assessment tools to comprehend self-harm behaviors is of utmost importance. Notably, the SAD PERSONS Scale has proven its efficacy in predicting hospital admissions and the length of stays after self-harm and suicide incidents. However, it had limitations in anticipating instances of repeated self-harm or suicide, leading to the recommendation that its routine use should be reconsidered (10).

Moreover, the crucial early identification of these behaviors is facilitated by the participation of experienced psychiatrists with diverse theoretical expertise. Their ability to integrate traditional psychoanalytic and contemporary mentalizing theories aids in comprehending self-harm and suicidal behaviors that have their origins in conflicted mental representations and compromised cognitive processing (Seng Choi).

To enhance our comprehension of self-harm behaviors spanning different generations, the Five Self-Harm Behavior Groupings Measure (5S-HM) offers a fresh and comprehensive assessment approach. By encompassing a broad spectrum of self-harm behaviors, even those typically overlooked, this tool offers a more complete view of self-harm (Liljedahl et al.). The empirical and thematic data gathered through this assessment offer valuable insights into the motivations and consequences of self-harm, transcending generational boundaries. It signifies a step toward a more nuanced approach to self-harm prevention and intervention.

## Management options

Addressing self-harm across age groups in the post-pandemic COVID-19 era necessitates tailored prevention strategies. Early identification and school-based mental health education are crucial for children and adolescents (11, 12). Young adults benefit from stress management programs, peer support, and workplace mental health initiatives. To combat the stigma associated with non-suicidal self-injury among adolescents, an adapted socio-ecological model recognizing factors across individual, family, interpersonal, institutional, community, and political levels can be applied. This revised model consolidates individual and interpersonal levels into the “individual and family” category while introducing evidence-based research as a fifth level (Thum et al.).

As adults, they require accessible mental health care, destigmatization efforts, and public awareness campaigns (13). For the elderly, particularly those experiencing isolation, it is crucial to undergo regular mental health assessments, participate in social engagement programs, and integrate a spiritual dimension into their care. This may include collaborating with religious entities, such as churches, as exemplified in a case report featuring an elderly patient from Malaysia who experienced depression and engaged in a suicide attempt (Mohd Salleh Sahimi et al.). Such partnerships can offer an extra channel for the early detection of mental health issues and, with appropriate training, facilitate the provision of professional counseling. For Muslims, the integration of the Islamic psychospiritual approach with psychotherapy in religious organizations is recommended as a culturally sensitive strategy for addressing self-harm (14). This approach not only attends to self-harm but also seeks to enhance individual spiritual development through incorporating Islamic rituals. It is important to acknowledge that the significance of this specific approach in suicide prevention is part of a broader range of interventions detailed in the Islamic psychological literature (14).

Additionally, ensuring widespread access to crisis helplines and intervention services is essential, alongside educational and awareness campaigns tailored for all age groups. Ultimately, the common goal in preventing self-harm behaviors across different ages is to nurture resilience and promote healthy coping skills.

In summary, understanding the prevalence, risk factors, and prevention of self-harm across age groups is crucial. This knowledge enables the creation of age-tailored prevention strategies, reduces stigmas, and promotes holistic mental health care. By refining individuals' unique needs at different life stages, we pave the way for a future where self-harm is demystified, and support and prevention efforts are maximally effective. The present case series enhances scientific understanding of the intricate link between pandemic-related stressors and self-harm behaviors, emphasizing the need for a comprehensive mental health approach during global crises like COVID-19.

## Author contributions

AJ: Conceptualization, Writing—original draft, Writing—review & editing. SM: Writing—review & editing. MR: Writing—review & editing. HL: Writing—review & editing.
